# Prevalence and Mortality of Influenza A (H1N1) Virus Among Patients With Acute Respiratory Infection in Southwest Iran

**DOI:** 10.5812/jjm.9263

**Published:** 2014-04-01

**Authors:** Seyed Mohammad Alavi, Roohangiz Nashibi, Fatemeh Moradpoor

**Affiliations:** 1 Health Research Institue, Infectious and Tropical Diseases Research Center, Ahvaz, IR Iran; 2 Infectious Diseases Department, Medical School, Ahvaz Jundishapur University of Medical Sciences, Ahvaz, IR Iran; 3 Health Institute, Ahvaz Jundishapur University of Medical Sciences, Ahvaz, IR Iran

**Keywords:** Influenza A Virus, H1N1 Subtype, Mortality, Epidemiology

## Abstract

**Background:**

Khuzestan and other parts of Iran were involved with Influenza A (H1N1) pandemic in 2009.

**Objectives:**

The aim of this study was to describe the prevalence and mortality of H1N1 in Behbahan, a city in Khuzestan, Southwest of Iran.

**Patients and Methods:**

The study population consisted of cases of influenza, hospitalized or referred to the city health centers. Diagnosis of H1N1 virus infection was based on rapid antigen testing (RT-PCR) of nasopharyngeal swabs. Data extracted from epidemiological survey forms, including demographic and clinical characteristics, laboratory results, risk factors and underlying diseases, medications, and treatment outcomes of patients were analyzed using SPSS 16 software by using Pearson chi-square test.

**Results:**

From a total of 318 patients, 180 (56.6%) were male and 138 (43.4%) female. Total number of patients with positive H1N1 tests was 167 (52.5%) with a male: female ratio of 1.2:1. Of total 318 admitted patients, 173 (96.1%) males and 135 (97.8%) females recovered and 10 people (7 (3.9%) males and 3 (2.3%) females) died, among which, three had positive test results for H1N1. The most prevalent signs and symptoms were: fever in 308 (96%) patients, cough in 278 (86.6%), lower respiratory symptoms in 208 (64.8%), gastrointestinal symptoms in 90 (28%), respiratory distress in 45 (13.7), and flu-like symptoms in 65 (20.2%) patients.

**Conclusions:**

Prevalence rate of H1N1 infection in the study region was higher compared to other part of Iran; but, close to the expected rate. The H1N1-associated mortality rate was lower than the reported rates in Iran and other parts of the world.

## 1. Background

Influenza is an acute and self-limited respiratory disease caused by influenza A and B viruses. During the past 400 years, influenza has caused a respiratory disease epidemic outbreak every 1 - 3 years. The largest influenza pandemic is recorded in the years 1918 and 1919, sacrificing 21 million people worldwide, during three waves (1). The Emergence of a new flu strain has resulted in serious concerns among health experts, because of its increased risk of spread in the community. A sample of the new flu strain is swine-origin H1N1 virus, transmitted to people through pandemic spread (2). This pandemic occurred in 2009 and spread in over 3 months through the whole world (3). 

H1NI human infections vary from an asymptomatic and uncomplicated upper respiratory tract infection to a dangerous condition of severe pneumonia with severe organ damage associated with exacerbation of the underlying disease (1). There are some other microbial agents, which can cause illnesses similar to influenza; thus, the diagnostic decision is based on the epidemiological and clinical findings and treatment of those at high risk of mortality should not be delayed until laboratory confirmation is obtained (1, 4). Epidemic influenza symptoms in uncomplicated cases include fever, cough, sore throat, stuffy/runny nose, muscle pain; gastrointestinal symptoms such as diarrhea, nausea, vomiting and dehydration. In some of elderly and immunosuppressed cases fever may not occur. In severe cases, influenza is associated with shortness of breath, tachypnea, hypoxia, central nervous system involvement, severe dehydration, organ failure, and septic shock (1-4).

A group of chronic diseases, including asthma, chronic obstructive pulmonary disease (COPD), chronic renal or liver failure, diabetes mellitus (DM), and heart and lung diseases, have been identified as risk factors for influenza. Other risk factors include pregnancy and ages above 65 or under 2 years (5-10). According to the national influenza guidelines, patients with severe illness, risk factors and underlying disease, should be considered as patients with high mortalities (11). In the H1N1 pandemic, information on epidemiological characteristics such as morbidity and mortality rate is required for planning future interventions such as immunization.

## 2. Objectives

We conducted this study to obtain the above mentioned information in the study region, Khuzestan, as an important geopolitical area of Iran.

## 3. Patients and Methods

In this study, all patients with signs and symptoms compatible with influenza during the H1N1 pandemic (2009) in Behbahan city, located in Khuzestan province, southwest Iran, were studied. The study population consisted of cases of influenza hospitalized or referred to health centers covering 200,000 people. Diagnosis was made by an infectious diseases subspecialist (one of the authors) as the regional responsible of national influenza survey. According to the influenza national guidelines, patients were admitted and interviewed, and epidemiological survey forms were completed. Throat gargle or throat swab specimens were obtained from the patients and sent to the reference Virology Laboratory of Medical School in Ahvaz (AJUMS) and a national reference lab in Tehran. 

Diagnosis of H1N1 virus infection was based on rapid antigen testing (RT-PCR) of nasopharyngeal secretions swaps or the levels of antibodies in the serum of patients. Data extracted from epidemiological survey forms, including demographic and clinical characteristics, laboratory results, risk factors, as well as underlying diseases, medications, and treatment outcomes of patients, were analyzed by SPSS (Statistical Package for Social Sciences) version 16 software for windows (SPSS Inc, Chicago, IL) using chi-square and Fisher's exact tests. P-values less than 0.05 were considered as significant.

## 4. Results

From a total of 318 patients, 180 (56.6%) were males and 138 (43.4%) were females (Table 1). The patients’ ages ranged from 1 month to 79 years, with mean and median of 26.4 ± 17.3 and 26 years, respectively. The total number of patients with positive H1N1 tests was 61 (19.2%). Of total seropositive patients, 33 (54.1%) were males and 28 (45.9%) were females. Means of ages in H1N1 infected and non-infected patients were 29.6 ± 18.9 and 25.9 ± 17.9 years, respectively. There were no significant differences in gender and age between H1N1-positive and negative patients (Table 1). 

Of total 318 admitted patients, 173 (96.1%) males and 135 (97.8%) females recovered; but, 7 (3.9%) males and 3 (2.3%) females died, among which, three patients (with mortality rate of 3/61, approximately 5%) had positive test results for H1N1. The mean age for death in H1N1 patients was 51.8 ± 27.1 years, significantly greater than that of the recovered cases, which was 27.5 ± 17.6 years (P = 0.02). Three of 61 (4.9%) H1N1-positive patients died because of respiratory failure. All H1N1-positive patients without underlying diseases recovered. No death was reported among H1N1-infected elderly or pregnant women. Underlying diseases in H1N1-positive and H1N1-negative patients are shown in Table 2. 

Prevalence of H1N1 infection in patients with underlying diseases was higher compared with ones without underlying diseases (Tables 3 and 4); but, mortality in H1N1-postive and H1N1-negative patients with underlying diseases was not significantly different (Table 5). Underlying diseases and comorbidities such as bronchial asthma, cardiovascular insufficiency, chronic lung diseases, immunosuppression and obesity, were significantly higher in H1N1-infected patients compared with non-infected ones (Table 4). The most prevalent signs and symptoms were: fever in 308 (96%), cough in 278 (86.6%) and sputum production in 80 (21%) patients. Other clinical findings including lower respiratory symptoms, gastrointestinal symptoms and flu-like symptoms are shown in Table 1. There was no difference between H1N1-positive and H1N1-negative patients for clinical findings, except for sputum production (Table 1). 

**Table 1.** Comparison of H1N1 Positivity in Patients Based on Demographic Characteristics and Clinical Symptoms ^a^

Variables	H1N1-Positive, n = 61 ^b^	H1N1-Negative, n = 257	P Value
**Age, y**			0.12
< 1	7 (11.5)	66 (25.7)	
1 - 5	19 (31.1)	60 (23.3)	
5 - 35	16 (26.2)	45 (17.5)	
35 - 65	13 (21.3)	76 (29.6)	
> 65	6 (9.8)	10 (3.9)	
**Gender**			0.66
Male	33 (54.1)	147 (57.2)	
Female	28 (45.9)	110 (42.8)	
**Cough**	52 (85.2)	226 (87.9)	0.52
**Fever**	59 (96.7)	249 (96.9)	1.0
**Gastrointestinal symptoms**	23 (37.7)	67 (26.1)	0.08
**Flu-like symptoms**	13 (21.3)	52 (20.2)	0.86
**Sputum ** ^**c**^	25 (40.9)	55 (21.4)	0.0008

^a^ Data are presented in No. (%).

^b^ Abbreviations: H1N1, Hemagglutinin type1 Neuraminidase type1

^c^ Statistically significant, P < 0.05.

**Table 2.** Prevalence of Influenza Subtype H1N1 Infection and Outcome of the Illness Among Patients With Acute Respiratory Infection ^a^, ^b^

Underlying Diseases	ARI Patients, No. (%)	H1N1-Positive, No. (%)	Recovered Patients, No. (%)	Died
**Without underlying diseases**	222 (69.8)	33 (10.4)	222 (69.8)	0
**With underlying diseases**	96 (30.2)	28 (8.8)	86 (27.0)	10 (3.1)
Cardiovascular	22 (6.9)	10 (3.1)	19 (6.0)	4 (1.3)
Bronchial asthma	29 (12.3)	11 (3.4)	39 (12.3)	0
COPD	7 (2.2)	0 (0.0)	7 (2.2)	0
Immunosuppressed	12 (3.8)	3 (0.9)	11 (3.4)	2 (0.6)
DM	13 (0.9)	1 (0.3)	10 (3.1)	3 (0.9)
Obesity	2 (0.6)	0	1 (0.3)	1 (0.3)
Pregnancy	11(3.4)	3 (0.9)	11 (3.4)	0

^a^ Abbreviations: ARI, Acute Respiratory Infection; COPD, chronic obstructive pulmonary disease; DM, diabetes mellitus; H1N1, Hemagglutinin type1 Neuraminidase type1

^b^ Data are presented in No. (%).

**Table 3.** Comparison of H1N1 Positivity in Patients With and Without Underlying Diseases ^a^, ^b^

	Without Underlying Diseases	With Underlying Diseases	P Value
**H1N1**			0.003
Positive	33 (14.6)	28 (29.2)	
Negative	192(85.4)	68 (70.8)	
Total	225 (100)	96 (100)	

^a^ Data are presented in No. (%).

^b^ Abbreviations: H1N1, Hemagglutinin type1 Neuraminidase type1

**Table 4.** Comparison of H1N1 Positivity in Patients With Underlying Comorbidities ^a^,^b^

Comorbidity	H1N1-Positive, n = 61	H1N1-Negative, n = 257	P Value
**Asthma ** ^**c**^	23 (37.7)	6 (2.3)	< 0.001
**COPD ** ^**c**^	5 (8.2)	2 (0.8)	0.001
**Immunosuppressed ** ^**c**^	8 (13.1)	4 (1.5)	0.0003
**Pregnancy**	4 (6.5)	7 (2.7)	0.23
**DM**	1 (1.6)	12 (4.6)	0.47
**Obesity ** ^**c**^	2 (3.3)	0	0.03
**Cardiovascular diseases ** ^**c**^	13 (21.3)	9 (3.05)	< 0.001

^a^ Abbreviations: H1N1, Hemagglutinin type1 Neuraminidase type1; COPD, chronic obstructive pulmonary disease; DM, diabetes mellitus.

^b^ Data are presented in No. (%).

^c^ statistically significant, P < 0.05.

**Table 5.** Comparison of Recovery and Death Among Patients With Underlying Diseases ^a^,^b^

	Recovered	Died	P Value
**H1N1**			0.60
Positive	25 (29.1)	3 (30)	
Negative	61 (70.9)	7 (70)	
Total	86 (100)	10 (100)	

^a^ Abbreviations: H1N1, Hemagglutinin type1 Neuraminidase type1.

^b^ Data are presented in No. (%).

## 5. Discussion

The emergence of a new swine-origin flu strain, H1N1 virus, causes serious concerns among health experts, because of its increased risk of pandemic spread and mortality. This pandemic occurred in 2009 and spread to Iran as well as the whole world. The present study revealed that approximately 20% of patients with acute respiratory symptoms during the time of H1N1 pandemic (2009) in the study region (Behbahan) were infected with influenza A, subtype H1N1. This finding was higher than the prevalence of this virus in Kashan, Iran (12) during the same timeperiod (7.8%) and similar to the expected rate in the country (1, 4). Based on estimations, it was expected for approximately 20% of the patients to be infected with H1N1, 1% - 2% of whom required hospitalization with 1% - 2% mortality rates. This high prevalence rate might be attributed to our samples, which were obtained only from patients with moderate or severe diseases, according to the regional health policies.

This study showed that underlying diseases or comorbidities played important roles in susceptibility to H1N1 infection. Patients with underlying diseases were two times more H1N1-infected than those without comorbidities (29.2% vs. 14.6%). Patients with comorbidities such as bronchial asthma, cardiovascular insufficiency, chronic lung diseases, immunosuppression and obesity, were the most prevalent condition associated ones with H1N1 infection. This finding was inconsistent with the previous studies (9-11, 13, 14). Other known comorbidities and risk factors such as diabetes mellitus, pregnancy and old age, were not statistically significant as well in H1N1-infected patient in this region, but should be considered in the H1N1 pandemic (9-11, 13, 14). For a better judgment, further multicentric studies are recommended. 

In this study, approximately 5% of positive patients for H1N1 died because of respiratory failure. The effect of H1N1 infection in this study as the main cause of death was not clear and should be considered with caution; we think that underlying diseases may play an important role in the deaths of H1N1-infected patients. Indeed, H1N1 infection in association with chronic morbidities such as diabetes mellitus, cardiovascular diseases and immunosuppression, increases the risk of death in patients. Our finding on mortality rate was greater compared to some reports such as one from Guatemala with 2.7%, but less than the rates reported from Iran and other parts of the world, which were 7% - 11% (2, 6-10, 12-14). 

Some factors may be attributed to these differences compared to other studies (except in Guatemala); physician skilled in managing the patients, knowledge of the population about H1N1 infection achieved through health educations provided by health experts in national programs against the disease, the availability of anti-viral drugs (such as Tamiflu) in the region (provided by Khuzestan Health Center) and awareness of the regional health network during this wave of H1N1 pandemic. This study also showed that underlying diseases play important roles in severity of the respiratory infection and Have poor prognosis on outcome of treatment. All patients who died in our study had at least two comorbidities. DM, cardiovascular insufficiency, chronic lung diseases and immunosuppression were the most prevalent underlying diseases associated with the deaths in our patients. These findings were in agreement with the medical literature and previous studies (1, 5, 8-10, 12, 14). 

In our study, males were at a higher risk of H1N1 infection than females (although statistically not significant). This finding means that gender might affect the H1N1 acquisition. This result was inconsistent with the previous studies (1, 12-14). The present study showed that older individuals were at a higher risk of H1N1 infection than younger ones (although statistically not significant). Mortality in the aged patients was higher than in younger patients. Therefore, elderly could be considered as an important risk factor for death in H1N1-infected patients with acute respiratory illnesses. All published investigation on H1N1 pandemics are consisted with this finding (1, 2, 5, 7-10, 14). 

Comparing the prevalence of H1N1 infection in this study with that of children (1 - 6 years old) previously published in Iran, indicated that age can be considered as a risk factor for H1N1 infection (15, 16). In this study, similar to the literature and previous studies, clinical spectrum of acute respiratory infection in H1N1-infected patients did not differ from non-infected cases. In fact, H1N1 infection cannot be estimated from clinical findings and virology testing should be performed to diagnose the infection (1-4, 8, 15, 16). 

In our study, some limitations and weaknesses should be considered. Our study population was restricted to the admitted patients in hospitals or health centers, which may be biased and result in the underestimation of influenza in the whole community. It was also restricted to one city of Khuzestan and may not represent the whole province. The other source of bias included the influenza subgroups when we restricted our study to H1N1; however, the other subgroups might have been included in our study by mistake. Future population-based studies are recommended. In conclusion, prevalence rate of H1N1 infection in the study region was higher compared to other parts of Iran, but in accordance with the expected rate. The H1N1-associated mortality rate was lower than the reported rates in Iran and some other parts of the world.
